# Rewriting protein fate: emerging strategies for targeted protein degradation and associated challenges

**DOI:** 10.1042/BST20250441

**Published:** 2026-11-11

**Authors:** Andrea Bertran-Mostazo, Rosa Barrio

**Affiliations:** Center for Cooperative Research in Bioscience (CIC bioGUNE), Basque Research and Technology Alliance (BRTA), 48160 Derio, Spain

**Keywords:** drug discovery and design, pharmacology, protein-protein interactions, proteostasis, ubiquitin ligases, ubiquitin proteasome system

## Abstract

Classical small-molecule drugs have traditionally relied on an occupancy-driven paradigm that requires tight and sustained binding to a well-defined pocket on the target protein. Targeted protein degradation (TPD) approaches have emerged to overcome the limitations of traditional pharmacology to promote the selective degradation of disease-associated proteins, harnessing the central proteostasis pathways in eukaryotic cells: the ubiquitin proteasome system and the autophagy-lysosomal machinery. Thus, TPD exploits the idea of reprogramming cellular proteostasis for therapeutic benefits, dictating the fate of the target protein by means of degrader molecules that induce proximity between a target protein and components of the degradation machineries. The present review gives an overview of the molecular mechanisms of cellular proteostasis and how they are exploited for drug discovery purposes through TPD strategies. We describe some of the most explored degrader modalities, such as molecular glues, proteolysis targeting chimeras, and lysosomal targeting chimeras, as well as new modalities of degraders, highlighting their major advantages and limitations, and discuss the challenges associated with their development, including ligand identification, target degradability, or the possibility for their rational design. Furthermore, we consider recent advances in the design of these degraders to potentially address those limitations. Together, these insights established TPD as a transformative pharmacological modality that revolutionized drug discovery campaigns since it expands the druggable proteome, offering numerous advantages over traditional drugs.

## Introduction: the ubiquitin code and proteostasis mechanisms

Cellular homeostasis is preserved through a dynamic balance between protein synthesis, folding, trafficking, and degradation. This equilibrium is predominantly safeguarded by the ubiquitin proteasome system (UPS), the major director of protein turnover in eukaryotic cells. The UPS controls proteostasis by a coordinated enzymatic cascade that ultimately conjugates ubiquitin molecules to target proteins, determining their stability, function, and fate, while contributing to protein quality control by eliminating misfolded or damaged proteins. Ubiquitin, through the posttranslational modification (PTM) of target proteins, or ubiquitination, is a versatile tag that encompasses many molecular signals, serving as a central regulatory axis in cell biology and, therefore, controlling a wide range of cellular processes, including proteasomal degradation, cell cycle progression, autophagy, signal transduction, DNA repair, and immune responses [1].

Protein ubiquitination is orchestrated by a hierarchical cascade of three enzymes, comprising an E1-activating enzyme, an E2-conjugating enzyme, and an E3-ligase enzyme. First, ubiquitin binds to the E1, in an ATP-dependent manner, which activates ubiquitin, enabling its transfer to the E2-conjugating enzyme. The E2, in turn, interacts with the E3, which, subsequently, mediates the final covalent linkage of ubiquitin to specific target proteins (Figure 1). Whereas limited numbers of E1 and E2 enzymes have been identified and described in humans, 2 and 40, respectively, there are more than 650 E3 ligases encoded in the human genome that recruit substrate proteins for ubiquitination, thus conferring specificity to the system [2,3]. The conjugated ubiquitin can be further modified in any of its seven lysine residues (K6, K11, K27, K29, K33, K48, K63) or in the N-terminal methionine (M1) to form homotypic or heterotypic ubiquitin chains. The diversity of the ubiquitin chains determines the divergent outcomes of substrate proteins, including proteasomal (K48 linkages) or autophagy-lysosomal degradation (K63 linkages), as well as other non-degradative effects. The activity of E3 ligases is counteracted by the action of the deubiquitinases (DUBs), which remove or edit the ubiquitin chains conjugated to the substrate proteins, conditioning protein fate [4]. The multiple possible types of ubiquitin chains are known as the ‘Ubiquitin code’ and dictate the substrate fate [5–7]. Some E3s are specific for other ubiquitin-like proteins (such as SUMO, NEDD8, or UFM1). Besides, the versatility of E3 ligases includes the transfer of ubiquitin to other amino acids besides lysines or N-terminal methionines in substrate proteins and, even more, to other non-protein substrates [2].

**Figure 1:**
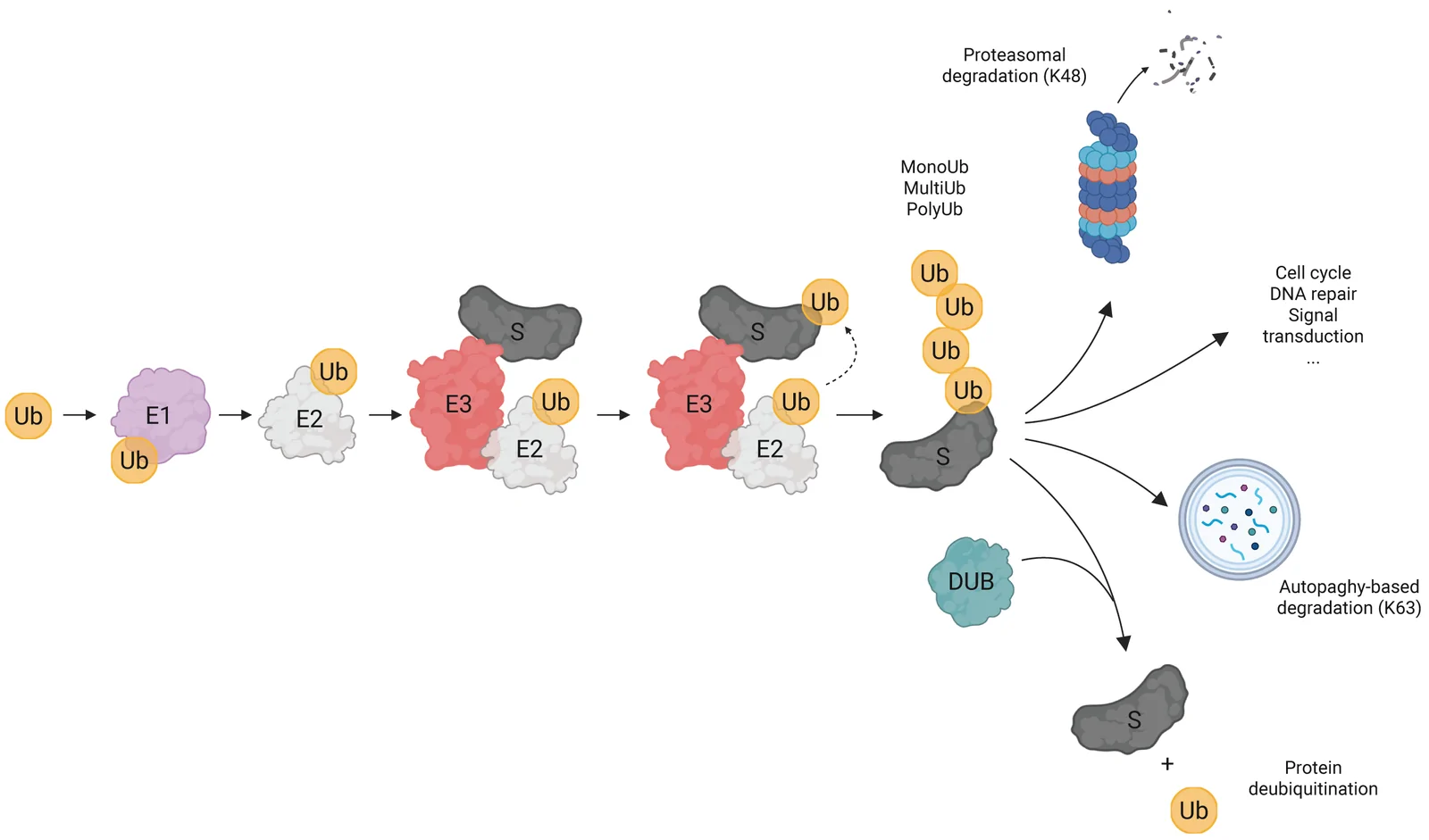
Schematic representation of the ubiquitination cascade A ubiquitin (Ub) molecule is activated through an E1-activating enzyme and then conjugated to an E2-conjugating enzyme. An E3 ligase recruits the protein substrate (S) and facilitates the transfer of the Ub to the target. Protein ubiquitination determines protein fate, depending on the length and type of Ub branches. This figure was created with Biorender.com.

E3 ligases belong to a very broad and versatile protein family, leading to a complex categorization. Nonetheless, E3s have been typically classified into three groups based on different structural domains and mechanistic traits [8,9]. The RING (really interesting new gene) group comprises around 600 E3 ligases, being the largest subfamily. They function as scaffold proteins, mediating the direct transfer of the ubiquitin from the E2 to the substrates. Among them, the complex Cullin-RING E3 ligases represent the major subclass (with about 400 members), which have a modular structure with multiple domains and require an extra layer of regulation arbitrated by the PTM by another ubiquitin-like NEDD8 or NEDDylation [10]. Conversely, the other two E3 groups, the HECT (homologous to E6AP C-terminus) and the RBR (Ring between RING) E3 ligases, catalyze the transfer of the ubiquitin molecule to the target in a two-step reaction process via the formation of a catalytic Ub∼E3 thioester intermediate prior to conjugation to the substrate protein. Whereas the HECT family comprises approximately 30 members, the RBR is the smallest with less than 20 [11]. Lastly, there is a set of E3 ligases that is classified as ‘atypical,’ since they display distinct conformational features, catalytic domains, and mechanisms of action from the other groups [9].

Considering that substrate specificity resides mainly in the E3s, their diversification, together with their strict regulation at multiple levels, ensures tight spatial and temporal control of protein substrate fate in eukaryotes. The activity of E3 ligases is not only adjusted by the DUBs but also by their expression levels and momentum in the organism, their subcellular co-localization with protein substrates, or their modification by diverse PTMs, such as phosphorylation and acetylation, as well as modifications by other ubiquitin-like proteins like NEDDylation or SUMOylation, that can influence the recognition of the substrate by the E3 or the type of ubiquitin chain assembled into the substrates [4–6].

In parallel, eukaryotic cells also rely on a complementary mechanism to supervise protein clearance and quality control. Particularly, under stress conditions, cells utilize the autophagy-lysosomal protein degradation pathway to remove long-lived and misfolded proteins, protein aggregates, and, in some cases, entire organelles. Protein degradation is mediated by multiple activated hydrolases contained in the lysosomes. First, proteins are sequestered into double-membrane vesicles named autophagosomes that are later fused with lysosomes, resulting in complete protein removal. Similar to proteasomal degradation, ubiquitin plays an important role in autophagy, since it serves as a signal to recruit autophagic receptors and to initiate autophagosome formation [12]. Hence, the importance of ubiquitin and ubiquitination is reinforced in cellular proteostasis to regulate protein fate.

## Targeted protein degradation: harnessing the ubiquitin signaling as a therapeutic modality

The concept that ubiquitin signaling determines proteins’ fate, propitiating distinct cellular outcomes, raised the possibility to transform proteolysis into a programmable system to redirect and specifically eliminate disease-associated proteins. In recent years, Targeted protein degradation (TPD) has emerged as a promising pharmacological approach that harnesses the degradative machinery of the cells seeking to mark specific target proteins for degradation, thus remodeling the intrinsic specificity and functions of the ubiquitin signaling into a therapeutic tool [13].

Classical drugs, which are mainly designed to modulate protein activity, operate through an occupancy-driven pharmacology and require tight and sustained binding of the drug in a well-defined binding pocket on the target to modulate its activity. In contrast, TPD approaches hijack the endogenous ubiquitin machinery of cells by inducing proximity between an E3 ligase and a protein of interest (POI) through a degrader molecule, triggering POI ubiquitination and consequent protein removal by the proteasome. Induced protein degradation is achieved through an event-driven mechanism that requires the formation of a ternary complex between the three partners implicated (E3:degrader molecule:POI), highlighting the relevance of E3 ligase and POI expression and colocalization for TPD approaches. Even though there are numerous classical inhibitors already approved in the clinic, there are still many disease-relevant proteins that remain pharmacologically intractable, as they lack well-defined binding pockets or possess scaffolding rather than enzymatic functions, making it difficult to address their modulation. In this context, TPD modalities introduce novel pharmacological possibilities and offer some advantages over classical drugs, enabling to tackle these intractable target proteins and thus expanding the pharmacologically accessible, or so-called *druggable*, proteome. First, since they work through an event-driven mechanism, they do not need tight binding between the drug and the target. Second, the target is degraded, not inhibited, resulting in the elimination of all the target functions, including the scaffolding ones. Third, and very interestingly, the target does not have to be saturated by the ligand to be degraded, so the degraders can work substoichiometrically and can be recycled once the target is eliminated, allowing lower doses of drug per treatment [13–15].

TPD strategies represent a paradigm shift over traditional drugs and have been recently consolidated as potential therapeutic tools to tackle *undruggable* proteins. Indeed, there are molecular glues (MGs) already approved in the clinic (IMiDs, i.e. lenalidomide or pomalidomide) and some proteolysis targeting chimera molecules (PROTACs), such as ARV-110, that are currently in Phase II clinical trials [16]. Crucially, on May 1st, 2026, the FDA approved VEPPANU (vepdegestrant, ARV-471), the first PROTAC molecule for the treatment of ESR1m, ER+/HER2-advanced breast cancer [17]. Degrader modalities are evolving quickly, from the above-mentioned initial MGs and PROTACs to lysosomal targeting chimeras (LYTACs), autophagy targeting chimeras (AUTACs), or hydrophobic tags (HyTs), among other strategies (Figure 2 and Table 1), which exploit the different degradative systems of eukaryotic cells, including not only the UPS but also the lysosomes or chaperone-dependent degradative pathways. Furthermore, TPD has also evolved towards other non-degradative induced-proximity strategies aiming to modulate the POI, proposing extra pharmacological opportunities, such as phosphorylation targeting chimeras (PhosTACs) or DUB targeting chimeras (DUBTACs) (Figure 3 and Table 1) [18]. In the present review we will present some of the most explored degradative strategies with some examples already in clinical phases, exploiting both the proteasomal and the autophagy-lysosomal degradative pathways and some relevant non-degradative strategies.

**Figure 2:**
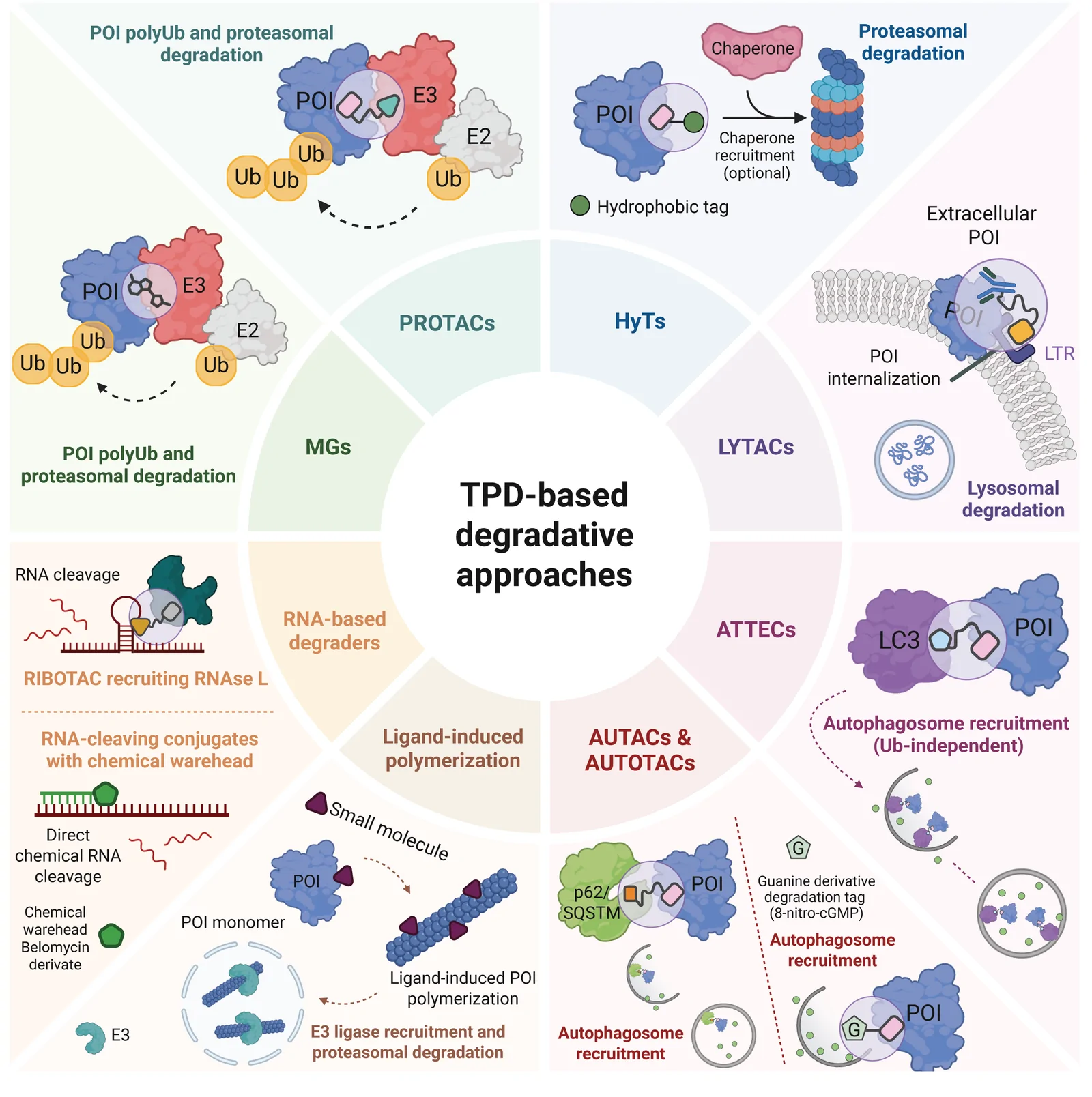
Graphical representation of the TPD-based degradative approaches described in this manuscript This figure was created with Biorender.com.

**Figure 3:**
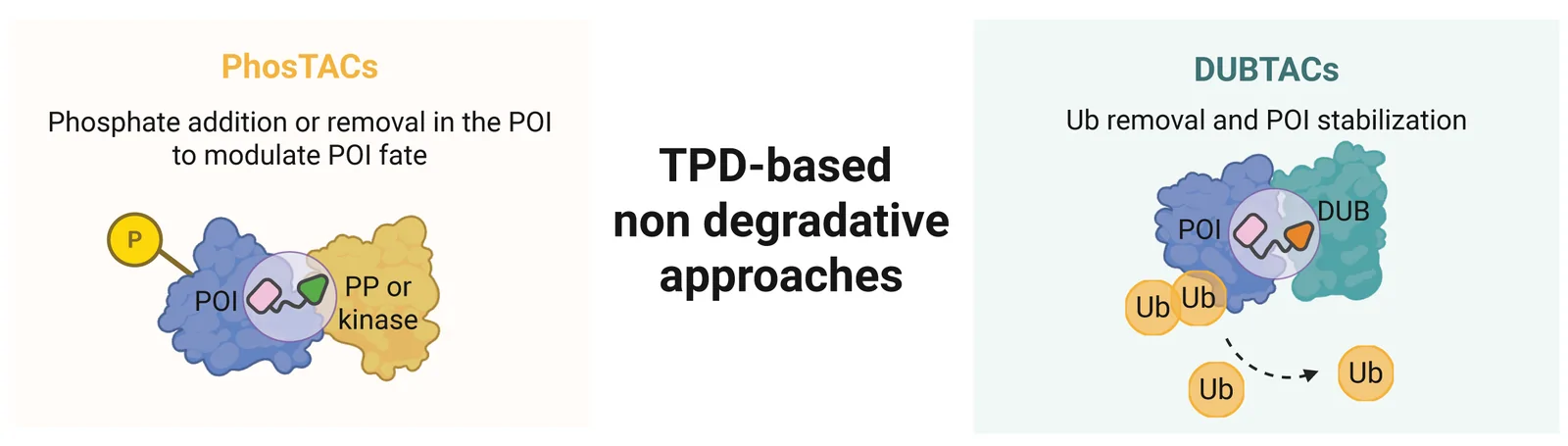
Graphical representation of the TPD-based non-degradative approaches described in this manuscript This figure was created with Biorender.com.

**Table 1:** Summary of degradative and non-degradative TPD-based strategies

	Strategy	Principle	Degradation pathway	Main advantage	Key limitation	Example
**Degradative strategies**	MGs	Monovalent degraders that induce and/or stabilize protein–protein interactions between an E3 ligase and a POI	Proteasomal	Small molecules (no linker required), high cooperativity	Challenging rational design for new molecules	IMiDs (i.e. lenalidomide, pomalidomide), and derivatives (i.e. CC-885) [19]
	PROTACs	Heterobifunctional degraders that interact with a POI and an E3 ligase, assembling a ternary complex that promotes POI ubiquitination	Proteasomal	Catalytic mechanism	High MW molecules, poor permeability, and poor drug-like profile. Limited number of ligands targeting E3s	ARV-110, ARV-471, dBET1 [20]
	HyTs	Hydrophobic tags that mimic unfolded proteins leading to protein degradation	Cellular quality-control system	No need for E3 ligase recruitment (E3 ligase agnostic)	Lack of selectivity due to pathway heterogeneity, risk of off-target proteotoxicity	SARD279 [21]
	Ligand-induced POI polymerization	Glue-induced POI polymerization to facilitate POI degradation	Proteasomal	Selective degradation of difficult and non-enzymatic targets. No need of bifunctional degraders or direct E3 recruitment	Exclusively depends on a suitable target architecture, small structural changes can abolish polymerization and degradation	BI-3802 [22]
	LYTACs	Heterobifunctional degraders that recruit an extracellular membrane POI to lysosomal-targeting receptors (LTRs)	Lysosomal	Enables degradation of extracellular proteins	Large and complex molecules with low tissue specificity	EGFR Ab-based LYTAC [23]
	AUTACs	Molecules that tag POIs with degradation signals (such as guanine-based tags) boost autophagic protein degradation	Autophagy-lysosomal	Can target protein aggregates and organelles	Unclear mechanism, potential lack of selectivity. Limited *in vivo* validation	MetAP2, FKBP12, BRD4, and mitochondria-targeting AUTACs [24]
	AUTOTACs	Heterobifunctional molecules directly recruiting autophagic receptors (p62/SQSTM1) to target proteins, promoting POI delivery to autophagosomes	Autophagy-lysosomal	Can target protein aggregates	Effect dependent on the expression levels of autophagic receptors	ATC161 targeting α-syn aggregates [25]
	ATTECs	Molecules that directly target POI to LC3 receptor, enabling POI sequestration into autophagosomes independent of ubiquitination	Autophagy-lysosomal	Ubiquitin-independent degradation	Potential off-targets due to autophagy activation	10O5, 8F20, AN1, and AN2 ATTECs targeting mHTT [26,27]
	RNA-based degraders	Target-RNA recognition to recruit an endogenous ribonuclease or a catalytic RNA-cleaving group to induce RNA cleavage	RNase-mediated pathways	Directly eliminate disease-associated RNA, rather than only inhibiting or degrading the encoded protein	Activity dependent on RNA structure, cleavage site accessibility, RNA abundance	pre-miR-96-targeting RiboTAC [28]
**Non-degradative strategies**	PhosTACs	Modulate POI phosphorylation to control POI localization, stability, and activity	-	Selective modulation of POI phosphorylation	Phosphorylation site accessibility and complex relation between phosphorylation and POI activity and stability	Tau-targeting PhosTAC [29]
	DUBTACs	Promote protein stabilization by preventing protein degradation upon Ub removal of POI	-	POI stabilization and POI function rescue	Efficacy might be dependent on POI ubiquitination state and cellular context	NJH-2-057 [30]

## Chemical strategies for targeted protein degradation

Based on the concept of exploiting the degradative machineries of eukaryotic cells, several strategies have been designed to chemically induce proximity between a target protein and an E3 ligase (proteasomal degradation-based strategies) or autophagy-lysosomal components (autophagy-lysosomal-based strategies), recruiting a specific component of each degradative machinery to induce selective POI removal.

### Molecular glues

MGs are small-molecule-based monovalent degraders. Being small molecules, they can comply with the ‘Rule of Five’ criteria, becoming appealing candidates for drug discovery purposes [31]. Particularly, MGs bind to the interface between an E3 ligase and a POI, acting like a ‘glue’ between the two partners, either reinforcing the complementarity on the interface of preexisting interactions or inducing and stabilizing non-native ones. The assembly of this ternary complex facilitates the ubiquitin transfer to the POI, triggering its ubiquitination and proteasomal degradation (Figure 2, MGs). Notably, in some cases, upon binding to the E3 ligase, MGs can reshape the substrate recognition site of the E3, altering the binding interface and leading to novel protein (neosubstrate) recruitment, thus broadening the potential of E3 ligase degradative capacity [32,33]. The first MGs described were cyclosporine A, FK506, and rapamycin, all natural products that were able to remodel their target proteins by inducing or stabilizing weak protein–protein interactions. A landmark case of MGs is the immunomodulatory drugs (IMiDs), i.e. thalidomide and its derivatives, that were previously approved as sedative and anti-nausea treatments for pregnant women but were later withdrawn from the clinic due to severe teratogenic effects. IMiDs were finally repurposed for the treatment of hematological malignancies since it was discovered that they were recruiting the CRBN E3 ligase to promote the ubiquitination and degradation of the transcription factors Ikaros and Aiolos [34,35], renewing the interest of IMiDs for cancer treatment. These findings established the foundation that MGs can stabilize protein–protein interactions to induce TPD, increasing the interest in the development of MGs as promising therapeutic opportunities [16,36]. Over the past years, MGs have been widely used in the clinic, but the rational design of MGs is still a huge challenge [16,36].

### Proteolysis targeting chimera molecules

PROTACs are one of the most explored TPD strategies. Indeed, there is a growing number of examples targeting clinically relevant proteins, such as estrogen and androgen receptors, that are showing good results in clinical phases, and remarkably, vepdegestrant has recently been approved by the FDA, evidencing their translational therapeutic potential [17]. PROTACs are heterobifunctional molecules that approximate an E3 ligase and a POI using two different warhead ligands connected through a linker. On one side, the ligand recruits a particular E3, and on the other side, the ligand binds to the POI, inducing proximity between the POI and the E3 ligase. The simultaneous engagement of both proteins with the degrader molecule enables the formation of the ternary complex (E3:PROTAC:POI), resulting in POI ubiquitination and subsequent degradation by the proteasome (Figure 2, PROTACs) [37,38].

Beyond the binding affinity of the individual ligands (binary complexes), the key determinant of the PROTAC activity is the assembly of the ternary complex. Crucially, the structure, conformation, stability, flexibility, and dynamics of the ternary complex dictate the resulting interaction between the POI and the E3 ligase and therefore define the degradative capacity of the molecule. This phenomenon is known as *cooperativity*. Indeed, the formation of a productive and efficient ternary complex is influenced by favorable and cooperative protein–protein interactions stabilizing and facilitating the assembly between the POI and the recruited E3 ligase. Therefore, the solely transient engagement of the complex is sufficient to promote POI ubiquitination and degradation. For this reason, PROTACs can act through a catalytic mechanism. Besides, PROTACs are not specifically designed to bind and inhibit the active site of the target protein. Indeed, they can be designed to target particular mutated residues associated with a disease or allosteric sites of the POI or the E3, allowing tight and selective spatial-temporal control of protein clearance. Nevertheless, despite their versatility, PROTACs present some limitations. Due to their natural chemical complexity, PROTACs offer the possibility to be rationally designed but require the identification of ligands binding to the POI and to the E3, respectively. This often results in large molecules with high molecular weight (MW), significantly affecting their permeability, oral bioavailability, and general physicochemical properties [39–42].

Interestingly, in recent years, antibacterial strategies have been developed based on the PROTAC strategy. The limited capacity to discover novel antibiotics, together with the continuous and rapidly increasing antibiotic resistance acquired by pathogens, led to the development of BacPROTACs. Taking advantage of the PROTACs technology, BacPROTACs have emerged as an attractive strategy to modulate protein degradation in bacteria by exploiting the microbial protein quality control system ClpC [43].

## Emerging targeted protein degradation strategies

Together, PROTACs and MGs have demonstrated how small-molecule-based drugs can remodel the ubiquitin signaling to induce selective degradation of intracellular proteins. The rapid advancement of TPD, together with some of its limitations, has leveraged its principles into novel strategies with alternative therapeutic potential.

### Lysosomal targeting chimera molecules

LYTACs exploit the concept of induced-proximity to achieve selective degradation of extracellular and membrane-associated POIs, which represent up to 40% of the human proteome [44]. Similarly to PROTACs, LYTACs are bifunctional chimeric molecules that connect the extracellular POI with a cell-surface lysosome-targeting receptor (LTR), promoting the internalization of the extracellular POI into lysosomes and its final lysosomal degradation (Figure 2, LYTACs). The binder of the POI can display several forms, such as small-molecule ligands, antibodies, or polypeptides. Moreover, a second generation of LYTACs has been developed, recruiting the asialoglycoprotein receptor, a liver-specific LTR, to degrade extracellular proteins in a cell-type-specific manner [23]. LYTACs suffer from the drug discovery challenges typical of large and high MW molecules, like instability upon administration and problematic delivery. Even though they are in early stages of development, and their mechanism of action is not totally understood, they represent a powerful opportunity to target extracellular proteins [44,45].

### Autophagy-based degradation strategies: AUTACs, ATTECs, and AUTOTACs

Since autophagy is another key proteostasis pathway controlling protein quality control in eukaryotic cells, several strategies have been developed exploiting the autophagy-lysosome pathway, extending the scope of induced-proximity strategies beyond the proteasome. Crucially, these approaches enable the removal of larger proteins or aggregates that are not accessible for proteasomal degradation. For this reason, they are being investigated to tackle neurodegenerative diseases by targeting, for instance, α-synuclein protein aggregates [46,47].

A range of distinct TPD modalities using the autophagic protein clearance mechanism have been developed, with small variations in their mechanism of action. AUTACs are constructed by a ligand that binds the POI and a guanine derivative, connected through a linker (Figure 2, AUTACs). This guanine derivative moiety mimics a ubiquitin-like modification and serves as a degradation tag that marks the POI to be sequestered into autophagosomes, which later fuse with lysosomes, finally triggering POI degradation by the lysosomal hydrolases [48]. In the case of the autophagy targeting chimeras (AUTOTACs), they recruit the POI to autophagic receptors, such as Sequestrome1/p62, to promote their delivery into the autophagosomes (Figure 2, AUTOTACs) [49]. By contrast, autophagy tethering compounds (ATTECs) act in a ubiquitin-independent manner by directly engaging POI to the LC3 receptor, facilitating the sequestration of the POI into autophagic vesicles (Figure 2, ATTECs) [26]. Despite these strategies differing in the mechanism to engage the autophagic machinery, they demonstrate the potential and the promising therapeutic benefits of induced-proximity modalities exploiting the autophagic-lysosomal machinery [50].

### Hydrophobic tags

HyTs represent an alternative approach to direct selective protein degradation through the UPS. These molecules consist of a ligand binding to a POI fused to small hydrophobic moieties that resemble a misfolded protein, directing the POI to the cellular quality-control system and finally inducing POI degradation. Their mechanism of action is not fully understood, and target protein degradation can be achieved through different pathways. Thus, POIs could be directly recognized by the 20S proteasome in a ubiquitin-independent manner or directed to the protein quality control systems by recruiting specific chaperones, including Hsp70/Hsp90 (Figure 2, HyTs) [51]. Compared with PROTACs, HyTs are small molecules with moderate MW and, therefore, better drug-like properties and broader applicability, including targeting proteins within the central nervous system. Notably, they do not rely on the recruitment of an E3 ligase, offering the opportunity of a reduced resistance mechanism associated with E3 ligase mutations or loss of function. Due to the multiple pathways that can be harnessed to achieve protein degradation, one of the main limitations of the HyTs is the lack of specificity, since the degradability efficiency cannot be fully predicted. In addition, the promiscuity of HyTs may induce non-specific hydrophobic interactions, leading to potential off-target toxicities. Ultimately, other HyTs’ associated challenges reside in the rational design of these molecules, including the linker optimization and the identification of novel HyTs [21,51,52].

### Ligand-induced protein polymerization

Ligand-induced protein polymerization exemplifies a distinct emerging TPD modality, in which protein degradation is achieved upon target self-association or polymerization rather than E3 ligase recruitment. In this case, a small molecule induces selective and reversible POI polymerization, resulting in POI sequestration in cellular foci and subsequent POI proteasomal degradation (Figure 2, ligand-induced polymerization). This approach was developed by Słabicki, M et al*.*, who described that the molecule BI-3802 was able to induce BCL-6 homodimer self-association leading to BCL-6 higher-order filaments. This assembly facilitates recognition by the non-cullin E3 ligase SIAH1, ultimately inducing BCL-6 proteasomal degradation [22].

### Non-protein degraders: RNA-targeted degraders

Beyond protein targets, TPD has extended to degrade RNA by coupling selected RNA transcripts to an endogenous ribonuclease or a catalytic RNA-cleaving moiety (Figure 2, RNA-based degraders). This induced-proximity enables the catalytic elimination of structured noncoding RNAs and disease-associated transcripts. RNA-based degraders comprise (i) RiboTACs that recruit RNase L, (ii) catalytic RNA-cleaving conjugates bearing warheads such as bleomycin derivatives, and (iii) imidazole- or other heterocycle-based degraders that promote localized ribonuclease activity [28,53].

## Emerging non-degradative strategies based on TPD

The landscape of induced-proximity pharmacology has been broadened to non-degradative strategies that modulate protein fate by altering protein ubiquitination, stabilization, localization, or phosphorylation. Rather than inducing protein clearance, they enable the restoration, activation, or functional rewiring of disease-relevant proteins.

### Phosphorylation targeting chimeras

PhoTACs are heterobifunctional molecules designed to induce proximity between a POI and a kinase or phosphatase, aiming to modulate POI phosphorylation status and therefore, controlling POI stability, localization, or activity rather than promoting protein degradation (Figure 3, PhosTACs). The limitations of this strategy embrace the accessibility of the phosphosites, as well as the formation and geometry of the ternary complex. A distinguished example is the small-molecule-based PhosTAC targeting Tau phosphorylation through phosphatase PP2A recruitment [29].

### Deubiquitinases targeting chimeras

DUBTACs are heterobifunctional molecules that engage a POI and a DUB to counteract POI proteasomal degradation by removing Ub chains from the POI. This ultimately stabilizes the POI and rescues its function without requiring new protein synthesis or direct interference with the degradative machinery (Figure 3, DUBTACs). Nonetheless, DUBTAC efficacy is limited to protein substrates undergoing ubiquitin-dependent proteasomal degradation and depends deeply on the availability and recruitment of a specific DUB [30].

## Challenges and future perspectives in TPD approaches

TPD approaches have emerged as plausible therapeutic tools to tackle currently *undruggable* proteins intractable by conventional therapies. Despite the numerous advantages of TPD over traditional drugs, already described in this manuscript, the development of TPD strategies as pharmacologically applicable drugs remains challenging. The efficiency of these degrader molecules relies on a combination of multiple chemical and biological factors, several of which represent the major challenges faced in the development of effective molecules [54].

### The need for new E3 ligases

The recruitment of an E3 ligase is a mandatory step during the action of PROTACs and MGs. For this reason, E3 ligases have centered much of the drug discovery efforts for the development of novel degraders. However, although there are more than 650 E3 ligases in humans, only a few have been employed for TPD purposes. Currently, about a dozen different E3s have been used, but the majority of degraders hijack von Hippel-Lindau and CRBN E3 ligases, evidencing a clear mismatch between the repertoire of targeted E3 ligases and the total number of E3 members. This can be explained by the complexity and diversity of E3 ligases and, importantly, the lack of specific small molecules targeting them [55]. The limited landscape of targeted E3s can contribute to the development of resistance mechanisms. Prolonged exposures to degrader molecules can ultimately alter the components involved in POI ubiquitination and degradation, for instance, provoking mutations or the down-regulation of the recruited E3. This points out the vulnerability of degrader strategies based on a limited set of E3 ligases. Besides, an expanded toolkit of recruitable E3 ligases could be advantageous for exploiting tissue specificity, since some POIs are involved in concrete specific diseases affecting particular tissues or organs. For these reasons, drug discovery efforts, mainly including chemoproteomic profiling, E3-focused fragment screening, and *in silico*-based E3 ligand identification, are nowadays centered on expanding the toolbox of E3 ligases that are being used for pharmacological approaches [56–58]. Recently, several examples have been reported targeting novel E3 ligases such as SOCS2 [59], KLHDC2 [60], DCAF1 [61], or FBW7 [62], E3 ligases that had been unharnessed until now.

### Identification of new ligands

The identification of suitable ligands is still a major hurdle for degrader design. Generally, PROTACs and other heterobifunctional molecules are constructed following a ‘plug-and-play’ modular approach, connecting each of the warheads with a linker. This results in large molecules with high MW and usually high lipophilicity, since they are designed to bind hydrophobic pockets on target proteins, and their lipophilic profile aims to boost their ability to penetrate cell membranes. As a consequence, most of these degraders disobey the ‘Rule of Five’ guidelines of drug-like molecules described by Lipinski for oral bioavailable drugs [31]. The physicochemical properties of these large molecules contribute to low cell permeability, poor solubility, poor metabolic stability, and questionable biodistribution, attributes that usually complicate drug development. Hence, medicinal chemistry efforts are focused on optimizing permeability and metabolic stability while preserving productive induced proximity. By contrast, MGs and HyTs are smaller molecules that easily comply with the Lipinski rules, offering optimal features to advance them towards the clinic [63,64].

### Lysine accessibility

Beyond the availability of E3 ligases, efficient POI degradation does not exclusively depend on the binding capacity of the degrader molecules to each of the proteins but mostly on structural and geometric properties that determine and allow efficient POI ubiquitination. The assembly of a stable and properly oriented ternary complex is a crucial step that governs POI degradation, since it will influence the accessible lysine residues on the POI amenable to be ubiquitinated. This illustrates the importance of structural conformation and dynamics of complexes, evidencing that not all proteins might be degraded and not all binders can be used to build degraders [14,65,66].

### The hook effect

In this context, one of the drawbacks of heterobifunctional degrader molecules is the hook effect phenomenon: under excessive degrader concentrations, the binding of each partner protein (E3 or POI) with the degrader molecule is saturated, enriching the formation of binary complexes. This impedes the arrangement of the ternary complex and therefore reduces the efficacy of the degrader. To circumvent the hook effect, a wide range of different concentrations must be assessed to determine the optimal therapeutic window where the ternary complex assembly and the efficiency of the degrader are maximized [63].

### Rationale design of degraders

The physicochemical properties of degraders are intrinsically related to the degrader design, but principles for effective degrader design remain elusive. To address these challenges, a growing number of multidisciplinary strategies are being investigated to produce optimized degraders. First, the identification of ligands relies mostly on structure-based approaches, combining computer-aided drug design and biophysical methods, high-throughput screenings, and chemoproteomics. However, the discovery, particularly of MGs, depended so far on serendipity, as it was the case for IMiDs, since the rational design of MGs requires a deep understanding of the protein–protein interface and the ligand-induced reshape of this interface. Notably, recent studies have reported some insights to underpin the molecular mechanisms of ligand-induced protein–protein interactions to achieve a stable and successful ternary complex, shedding light to shift from a serendipitous discovery to a rational design of MGs [67–69]. Bifunctional degraders are also subjected to exhaustive investigation for further design optimization. Due to their natural modular composition, they are more amenable to be rationally designed than MGs. Beyond the ligands binding to each of the protein partners, the linker connecting both ligands represents a crucial element for an optimal configuration of the ternary complex. Linker length, composition, and rigidity can truly influence the final ternary complex, as well as the physicochemical properties and, therefore, the efficiency of the molecules [70,71]. Current approaches are exploring compacted ligands and conformational flexibility restrictions using rigid linkers or macrocyclic designs. It is obvious then that even small changes in the architecture of the molecules can lead to remarkable effects on the ternary complex assembly, cooperativity, and final POI degradation outcomes. In this context, artificial intelligence and machine learning (AI/ML) methods are being widely exploited to define and design successful degraders, not only by facilitating ligand identification but also by helping in predicting the physicochemical properties of the molecules, the assembly and stability of the ternary complex, and potential structure–activity relationships. Together, advances in computational methods, including AI/ML, chemoproteomics, and high-throughput screenings, are transforming the discovery of novel degraders [72–76].

### Beyond protein degradation

Besides TPD strategies harnessing the UPS, a growing number of degradative approaches are being investigated to expand the scope of TPD and induced-proximity strategies, such as the mentioned LYTACs and autophagy-based degraders that exploit the lysosomes and autophagy-lysosomal machineries. Moreover, novel TPD strategies evolved to degrade non-protein biomolecules, such as RNA, by approximating RNA molecules to specific ribonucleases to promote RNA cleavage [77], and the expansion to non-degradative induced-proximity strategies to modulate POI function, stability, or localization, instead of achieving complete protein removal [78]. In this context, regulated induced proximity targeting chimeras (RIPTACs) have emerged as a promising therapeutic strategy to tackle diseases, such as cancer, in a very selective manner. RIPTACs are heterobifunctional molecules that induce chemical proximity between a POI, highly expressed in tumor cells, and a pan-expressed protein essential for cell survival. The resulting ternary complex abolishes the function of the essential protein, thus inducing cell death, primarily in the context of cells with high expression of the POI [79].

Together, the advances in the TPD field and the emergence of innovative induced-proximity strategies are broadening the range of biomolecules that can be pharmacologically targeted, thus tackling the unmet clinical needs of society. Owing to the advantages offered, TPD-based approaches are becoming consolidated as an emerging but promising strategy in drug discovery campaigns. The results of the TPD-based molecules in clinical trials are promising and clearly demonstrate the change in paradigm in drug discovery, probing that small molecules can be used to reprogram the spatial and temporal localization, function, and fate of biomolecules within the cell in the organismal context.

## Perspectives

### Highlight the importance of the field

TPD has emerged as a transformative therapeutic modality that has shown a rapid and continuous growing interest of scientists, especially in the field of drug development and chemical biology. TPD exploits the endogenous proteostasis pathways to eliminate, rather than only inhibiting, specific target proteins. TPD works through an event-driven pharmacology instead of the occupancy-driven one that has dominated small-molecule pharmacology for decades. This offers novel possibilities and advantages over traditional drugs and, therefore, sheds light on targeting challenging proteins that still remain *undruggable*, including transcription factors, scaffolding proteins, or aggregation-prone proteins. Remarkably, on May 1st, the FDA approved the first-ever approved PROTAC-based degrader molecule for the treatment of breast cancer, demonstrating the therapeutic potential of these novel drugs. Furthermore, the degrader strategies have expanded beyond the UPS towards the autophagy-lysosomal pathway and to other non-degradative str ategies, highlighting the versatility of induced-proximity pharmacology to control and modulate protein fate.

### Provide a summary of the current thinking

The continuous advancements in the field demonstrate the potential of TPD to reprogram cellular degradation pathways to induce specific target protein degradation. The scope of TPD is no longer limited to PROTACs and MGs, but it has been broadened to distinct degrader and non-degrader modalities that exploit different principles to modulate protein fate. Each of these novel technologies presents unique opportunities and limitations that are continuously being investigated. All of them differ in the recruited cell machinery, the mechanistic requirements, and the obligated developmental process. Therefore, the established design principles for one molecule cannot be easily adapted or transferred to others. Currently, most efforts are made to undergo from the empirical or serendipitous discovery to a rational design of these molecules. For this reason, structural biology, AI/ML, or chemoproteomics are being applied to identify insights and determinants of key properties for degrader design, such as ligand identification, target degradability, or ternary complex formation. Nevertheless, the general framework for predictive design has not been yet accomplished. In this context, chemoproteomic platforms and covalent-fragment libraries are useful, since they enable the identification of ligandable pockets and reactive residues without the need for previous structural information. However, the translation of ligand identification to efficient protein degradation is not direct, since the prediction of the dynamics of the ternary complex is still unresolved. The interaction of three different partners considering the geometry and conformation of the assembly as well as the intracellular context is not easily anticipated. Computational tools are being increasingly applied but are still limited to uncomplete structural data and the vulnerability of AI/ML methods due to small and biased training sets, unreliable data, weak target degradability prediction, or poor extrapolation into real biological contexts. Thus, although these tools are helpful, the development of these molecules still demands experimental validation. The suboptimal pharmacokinetic properties of these molecules are also still a major constraint. Therefore, the ADMET predictions (absorption, distribution, metabolism, excretion, and toxicity) must be early incorporated and continuously tested in the analyses of these molecules. In this context, the development of prodrugs or targeted delivery of these molecules is being investigated. Despite the mentioned drawbacks of induced-proximity approaches, they have demonstrated enormous pharmacological potential.

### Comment on future directions

In line with induced-proximity modalities, it is important to remark that efforts are being made to broaden TPD approaches, not only to other non-degrative approaches to modulate protein fate, but also to specifically target other biomolecules such as DNA, RNA, or other biomolecules, such as glycans (DGlyTACs) [80] or protein methylations (MrTACs) [81]. Degrader-Antibody Conjugates [82] have also emerged as an attractive idea, since they combine the antigen-targeting precision of monoclonal antibodies with the degradative capacity of degraders, enabling the delivery of highly lipophilic degraders directly to tumor cells to maximize therapeutic index while eliminating off-target toxicities. Besides expanding the scope of TPD, enlarging the range of accessible targets for these degrader molecules, as well as the number of E3 ligases harnessed, is still a need that must be addressed. In this context, progress is being made in predicting target protein degradability or the recruitment of novel effectors, like unharnessed E3s. Besides, advances in AI/ML and structure-guided modelling are expected to facilitate and accelerate the rational design of these molecules while improving their properties and their biological outcomes. Together, this suggests that the field is evolving towards a more general concept of programmable pharmacology to manipulate cellular function for therapeutic purposes and to demonstrate the possibilities to expand the *druggable* proteome.

## Open Access

Open access for this article is provided via our Subscribe to Open programme, made possible through the support of our subscribing institutions. Learn more here: https://portlandpress.com/pages/open_access_options_and_prices#conditional.

## Abbreviations

AI/ML: artificial intelligence and machine learning
AUTACs: autophagy targeting chimeras
ATTECs: autophagy tethering compounds
DUBs: deubiquitinases
DUBTACs: DUB targeting chimeras
HECT: homologous to E6AP C-terminus
HyTs: hydrophobic tags
IMiDs: immunomodulatory drugs
LTR: lysosome-targeting receptor
MGs: molecular glues
MW: molecular weight
PhosTACs: phosphorylation targeting chimeras
PTM: posttranslational modification
PROTACs: proteolysis targeting chimera molecules
RIPTACs: regulated induced proximity targeting chimeras
RBR: RING between RING
RING: really interesting new gene
TPD: targeted protein degradation
UPS: ubiquitin proteasome system
